# High seebeck coefficient in middle-temperature thermocell with deep eutectic solvent

**DOI:** 10.1038/s41598-021-91419-5

**Published:** 2021-06-07

**Authors:** Naura Fakhira Antariksa, Teppei Yamada, Nobuo Kimizuka

**Affiliations:** 1 Division of Applied Chemistry, Graduate School of Engineering, Kyushu University, Motooka 744, Nishi-ku, Fukuoka, 819-0395 Japan; 2 Center for Molecular Systems, Kyushu University, Fukuoka, Japan; 3 Department of Chemistry, Graduate School of Science, The University of Tokyo, 7-3-1 Hongo, Bunkyo-ku, Tokyo, 113-0033 Japan

**Keywords:** Electrochemistry, Energy, Ionic liquids, Renewable energy

## Abstract

Deep eutectic solvent (DES) was applied to the solvent of thermocell and high Seebeck coefficient (*S*_e_) of the thermocell was achieved at high-temperatures operation. The *S*_e_ of a redox couple of ferricyanide and ferrocyanide ([Fe(CN)_6_]^3−/4−^) reaches − 1.67 mV/K in a DES consisting of ethylene glycol and choline chloride. Spectroscopic analysis reveals that this is due to the strong interactions between the redox couple and the DES. Furthermore, the cell can operate over a wide temperature range of 135–165 °C. This result is a desired feature for waste-heat recovery applications.

## Introduction

Approximately 60% of energy produced is lost as waste heat^1^, and thus harvesting this thermal energy is crucial in order to combat the energy crisis. In particular, middle-temperature waste heat in the range of 100–200 °C is available in abundance from the industrial sector, yet it is often wasted without any effort at recovery^2^. Thermoelectric conversion is an attractive method for waste heat harvesting, and the use of thermocells is a promising option due to their high Seebeck coefficients (*S*_*e*_)^3^. Similar to the *S*_*e*_ for solid-state thermoelectrical technologies, the *S*_*e*_ of thermocells is defined as the potential difference generated across a cell with a temperature gradient. In liquid-based systems, such a temperature dependence is also related to the redox reaction entropy of a given redox couple, yielding the following relationship:

1 $$Se = \frac{\Delta E}{{\Delta T}} = \frac{{\Delta S_{rc} }}{nF}$$

where *ΔE* is the open-circuit potential, *ΔT* is the temperature difference, *ΔS* is the partial molar entropy difference of the redox species (redox reaction entropy), *n* is the number of electrons in the reaction, and *F* is the Faraday constant^4,5^. *S*_*e*_ is one of the key parameters that determines the figure-of-merit of a thermocell, *ZT* = *S*_*e*_*Tσ/κ*, where *σ* is the electrical (or ionic) conductivity of the electrolyte, *κ* is the thermal conductivity, and *T* is the absolute temperature. The *ZT* value represents the efficiency of a thermocell and thus, high *S*_*e*_ and *σ* combined with a low *κ* are imperative in the design of a liquid-based thermo-electrochemical system. In addition to these requirements, the thermocell electrolyte for middle-temperature heat recovery necessitates excellent thermal stability at temperatures as high as ca. 200 °C. For this reason, thermocell systems utilizing ionic liquids (ILs) or high-boiling point organic solvents, such as MPN, DMSO, and γ-butyrolactone has been developed^6–13^. These solvents were found to have high thermal stability and negligible volatility at that temperature, making them suitable for middle-temperature waste-heat harvesting^14,15^. Furthermore, the wide range of operating-temperature of these solvents allows a large temperature difference (*ΔT*) to be applied to the thermocell, leading to a high power performance of the cell.

Despite their favorable thermal stability, some IL thermocells have been found to have lower *S*_*e*_ than their aqueous counterparts (Table S2), which stems from the small redox reaction entropy (*ΔS*). As shown in Eq. (1), the *S*_*e*_ of a thermocell is proportional to *ΔS*, meaning that a high *S*_*e*_ is a direct reflection of a large entropy change through the redox reaction. Previous studies^6,11^ suggested that the entropy change of a thermocell is related to the redox-mediated reorientation of solvent molecules. The reorientation process is governed by the “internal order” of a given solvent, which expresses the degree of organization of solvent molecules in the bulk state^13,16^. Solvents with low internal order can undergo reorientation with a small external perturbation, meaning that a large *ΔS,* and accordingly, a high *S*_*e*_ is expected. ILs, however, are known to be highly structured solvents^10,17^. As a result, the redox reaction entropy in IL medium is small, which limits the enhancement of *S*_*e*_. Therefore, it is crucial to develop a thermocell electrolyte that has both a high redox reaction entropy and wide operating-temperature.

**Figure 1:**
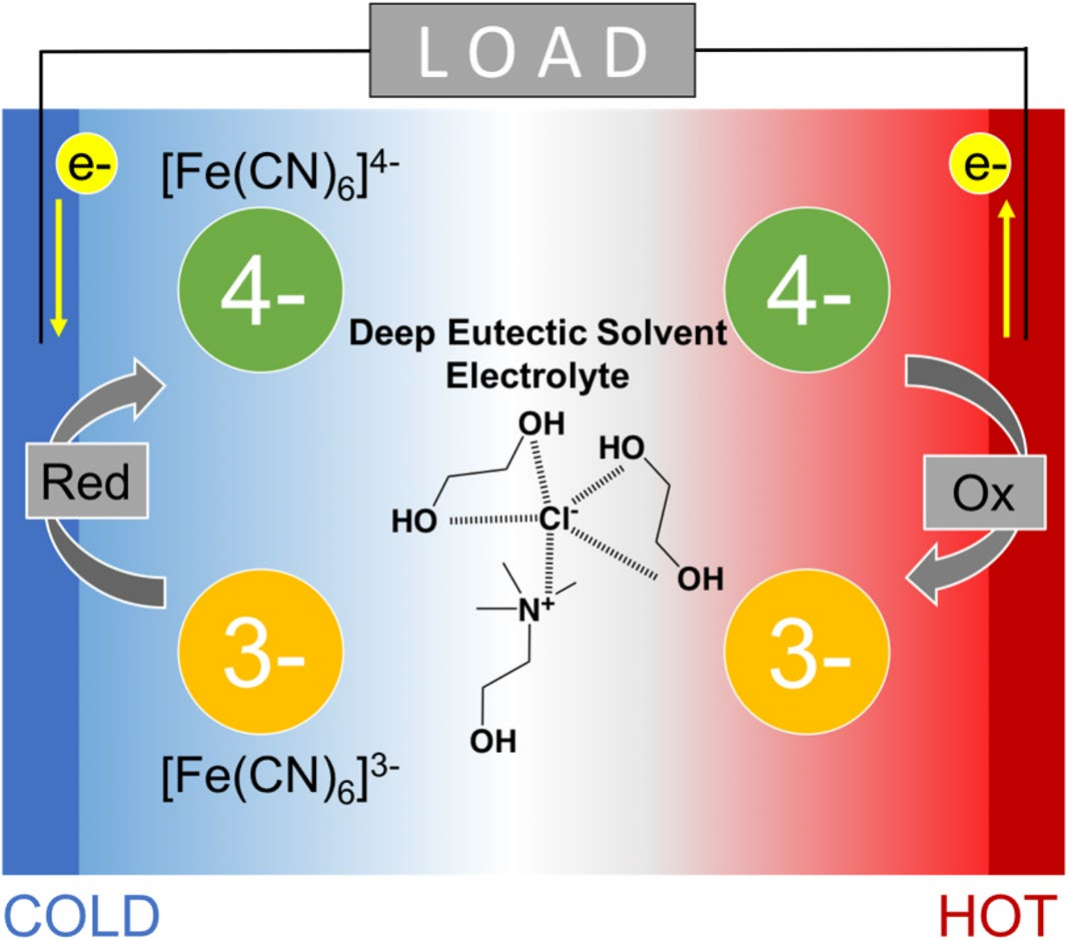
Schematic illustration of a [Fe(CN)_6_]^4−/3−^ thermocell, which utilizes DES ethaline as an electrolyte. On the hot side, an entropy-driven reaction, oxidation of [Fe(CN)_6_]^4−^ to [Fe(CN)_6_]^3−^, occurs. Conversely, on the cold side, an enthalpy-driven reduction reaction is promoted. This mechanism generates a potential difference across the cell, and thus electric energy can be harvested. Ethaline displays thermal stability in wide-temperature ranges, allowing this thermocell to operate at high temperatures. Moreover, the large change of solvation enthalpy resulted in the high Seebeck coefficient.

In this work, we propose a novel thermocell system that displays both a high S_e_ and the ability to operate at the middle-temperature range by the use of a deep eutectic solvent (DES) as an electrolyte. DES is a eutectic mixture of organic salt and a hydrogen-bond donor (Fig. 1). The melting point of such a mixture is significantly lower than either of its components^18,19^. The low melting point is caused by the complexation of the hydrogen bond donor with ionic species, which promotes the delocalization of ionic charges in the complex. This phenomenon leads to the lowering of the melting point of the mixture, and thus DESs exist as a liquid at room temperature. The physical properties of bulk DESs are similar to those of ILs, particularly in terms of their thermal stability^19^, while it has yet to be utilized for a thermocell. We chose to utilize one of the DESs known as ethaline, which comprises of a 1:2 mixture of choline chloride (ChCl) and ethylene glycol (EG). Ethaline was chosen due to its disorganized characteristic, which owes to the presence of the hydroxy constituent^20,21^. We envisioned that this characteristic corresponds to the low internal order of the ethaline DES, allowing a for large entropy change to arise and thus a high *S*_*e*_ to be obtained. Furthermore, a previous computational study on ionic liquids predicts that small, symmetrical, unbranched cation and anion lead to an increase in *S*_*e*_ value^22^. Considering that DES and ILs are comprised of similar species, we expect that the prediction mentioned above can be extrapolated to this DES system. Here, ethaline is constituted of relatively small and unbranched cation, Ch^+^, and hard base anion, Cl^−^, which would supposedly lead to a high *S*_*e*_. Importantly, the thermal stability of ethaline over a wide range (− 68 to 210 °C)^19^ would allow for the operation of thermocell at the desired middle-temperature range.

## Results and discussion

**Figure 2:**
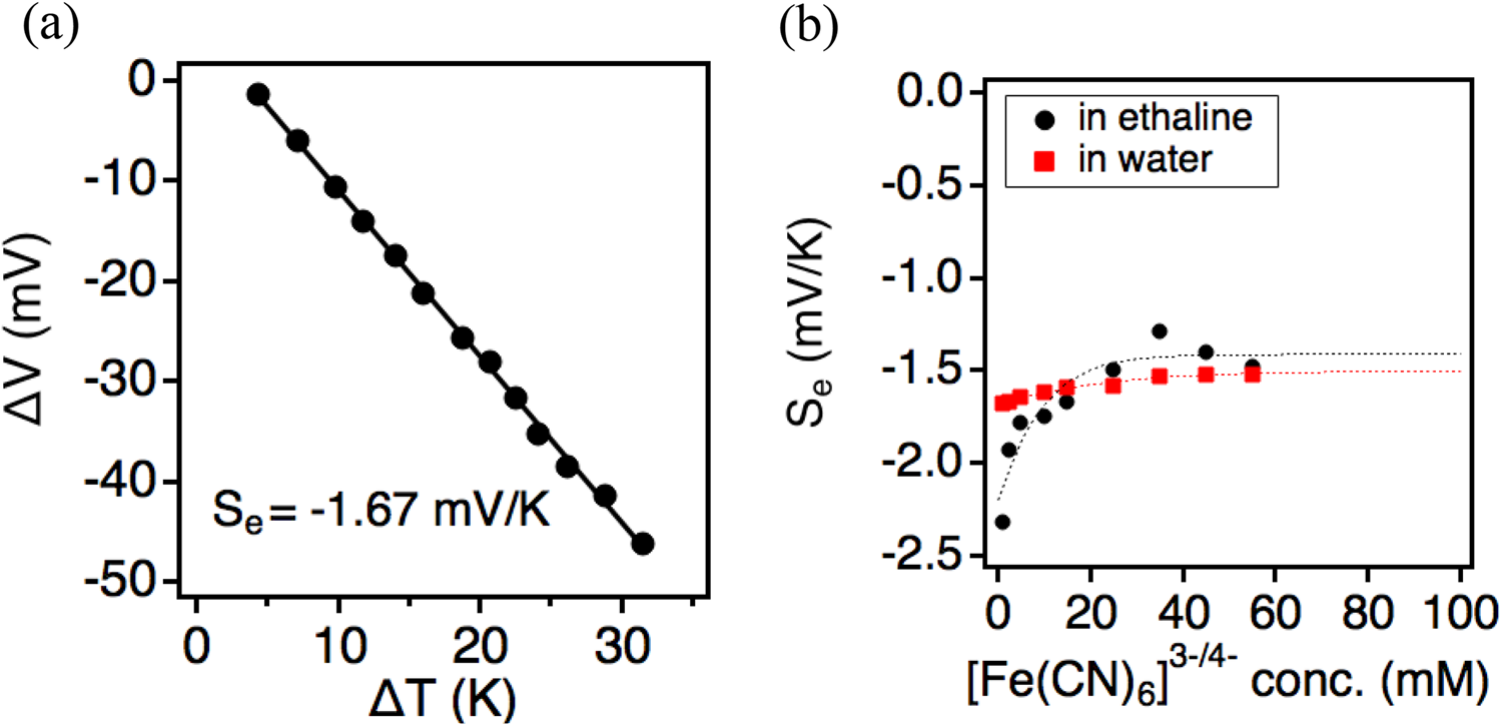
Seebeck coefficient of the ethaline thermocell. (**a**) The plot of open-circuit voltage against the temperature difference of the thermocell consisting of ethaline as a solvent and 15 mM of the redox species [Fe(CN)_6_]^4−/3−^ (*T*_*hot*_ = ca. 165 °C). (**b**) The concentration dependency of *S*_*e*_ of the ethaline thermocell (black circles) and aqueous thermocell (red squares).

*S*_*e*_ measurement of the ethaline thermocell was elaborated (detail of the experiment is shown in the Supporting Information). Figure 2a shows the open-circuit potential (*V*_*oc*_) of the cell plotted against temperature difference. The *S*_*e*_ of the thermocell was estimated from the slope of this plot to be − 1.67 mV K^−1^ with 15 mM of [Fe(CN)_6_]^3−^ and [Fe(CN)_6_]^4−^.

The high *S*_*e*_ of the ethaline thermocell can be attributed to the large entropy difference, specifically solvation entropy, between [Fe(CN)_6_]^3−^ and [Fe(CN)_6_]^4−^. [Fe(CN)_6_]^4−^ has a higher surface charge density compared to that of [Fe(CN)_6_]^3−^, which further regulates the orientation of solvent molecules^4,23,24^. The solvation effect of these redox species was investigated by FT-IR spectroscopy (Fig. S3a). [Fe(CN)_6_]^4−^ shows a broader C≡N stretching vibration peak with a full width at half maximum (FWHM) of 22.5 cm^−1^ against [Fe(CN)_6_]^3−^ with an FWHM of 9.8 cm^−1^. The broader peak corresponds to more substantial structural heterogeneity in the solvation environment of [Fe(CN)_6_]^4−^, which is originated from the stronger hydrogen bond interaction between the solute and the solvent^23,24^. This enhanced interaction, in turn, leads to a tightly-packed solvation shell surrounding [Fe(CN)_6_]^4−^ than that of [Fe(CN)_6_]^3−^. As a result, a large entropy difference presents between the two species, which is reflected in the high *S*_*e*_ of the ethaline thermocell.

FT-IR results also reveal the difference in the solvation effect between ethaline and water. The FWHM of [Fe(CN)_6_]^4−^ and [Fe(CN)_6_]^3−^ in water are 12.6 cm^−1^ and 7.4 cm^−1^, respectively (Fig. S3b). The difference in FWHM in water (5.2 cm^−1^) is inferior to that in ethaline (12.5 cm^−1^). The enhanced difference of FWHM shown in ethaline can be attributed to the exaggerated solvation interaction in ethaline compared to water, and thus the *S*_*e*_ of [Fe(CN)_6_]^3−/4−^ is higher in the former. This is in good agreement with the smaller difference of FWHM between [Fe(CN)_6_]^4−^ and [Fe(CN)_6_]^3−^, (4.8 cm^−1^) in an ionic liquid, [C_2_mim][EtSO_4_] (Fig. S3c), which shows lower *S*_*e*_.

The concentration dependency of *S*_*e*_ is shown in Fig. 2b. The absolute *S*_e_ value of the ethaline thermocell shows a gradual decrease with increasing the concentration of solutes. The *S*_*e*_ trend can be rationalized as an effect of increased electrostatic interaction as explained in the Debye–Hückel theory, where the *S*_*e*_ is proportional to the square root of electrolyte concentration^12,25,26^. The *S*_e_ trend observed in ethaline thermocell is similar to those observed in previously reported aqueous and ionic liquid thermocells^12,26,27^. However, the concentration dependency of *S*_*e*_ was more significant in ethaline thermocell than that in aqueous thermocell (Fig. 2b). This suggests that ethaline presents a dissimilar solvation environment to that in the aqueous electrolyte.

**Figure 3:**
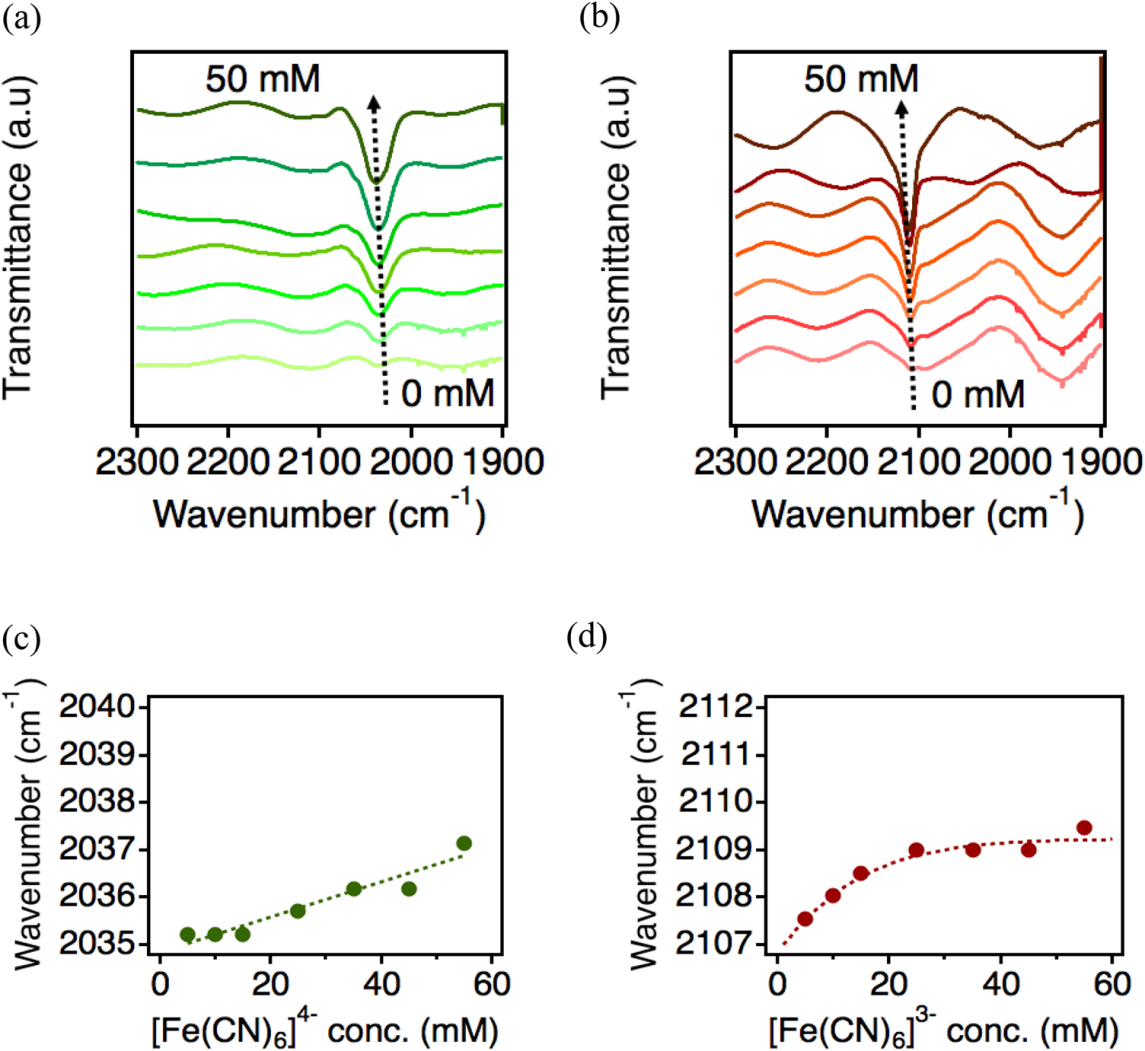
The FT-IR spectra of various concentrations of [Fe(CN)_6_]^4−^ (**a**) and [Fe(CN)_6_]^3−^ (**b**) in ethaline. C≡N stretching modes of the of [Fe(CN)_6_]^4−^ and [Fe(CN)_6_]^3−^ were fitted using the pseudo-Voight function^29^ and the peak wavenumbers were plotted in (**c**) and (**d**), respectively.

The solvation environment of various concentrations of [Fe(CN)_6_]^3−/4−^ in ethaline medium was further investigated by FT-IR spectroscopy (Fig. 3). C≡N stretching vibration is sensitive to the changes in electrostatic and H-bond environments, and so examining its behavior in different solute concentrations can give an illustration of the solvation structures surrounding [Fe(CN)_6_]^3−/4−^^28,29^. We speculate that the concentration of the redox species may affect the extent of H-bond formed between solvent–solute, which confirms the trend of *S*_*e*_. In the ethaline electrolyte (Fig. 3a,b), the increase of [Fe(CN)_6_]^3−/4−^ concentrations causes the blue-shift of C≡N stretching peaks. The blue-shift can originate from two factors; one is the intensity of the H-bond between solvent molecules and the nitrile ligand, and the other is the formation of an additional H-bond. These phenomena are termed as improper H-bonding, meaning that the strengthening or formation of the H-bond will cause the tightening of the C≡N bond, and thus the blue-shift^30–32^. By considering this, the increase of the [Fe(CN)_6_]^3−/4−^ concentration increases the number of H-bonds formed between ethaline and [Fe(CN)_6_]^3−/4−^. In contrast, a negligible shift was observed in the aqueous electrolyte (Fig. S4a and S4b), which implies that the amount of H-bond between the redox species and water is not affected by the concentration of solute. The spectral results show that ethaline is more sensitive to concentration changes compared to water. In other words, the introduction of [Fe(CN)_6_]^3−/4−^ more easily disrupts the hydrogen bond network of ethaline, and this allows the hydrogen bond donor, ethylene glycol, to interact readily with the solutes^33^. Ethaline consists of various functional groups such as hydroxyl groups of ethylene glycol, choline, and trimethylammonium groups, and they form various kinds of solvent–solute interaction that may, in turn, alter the solvation structure surrounding the redox couple. Since H-bond formation happens more readily in ethaline, the alteration is likely to be more significant in this solvent compared to water. As a result, the *S*_*e*_ of [Fe(CN)_6_]^3−/4−^ undergoes more prominent changes with differing concentrations of the redox couple, which reflects the *S*_*e*_ trend shown in Fig. 2b.

**Figure 4:**
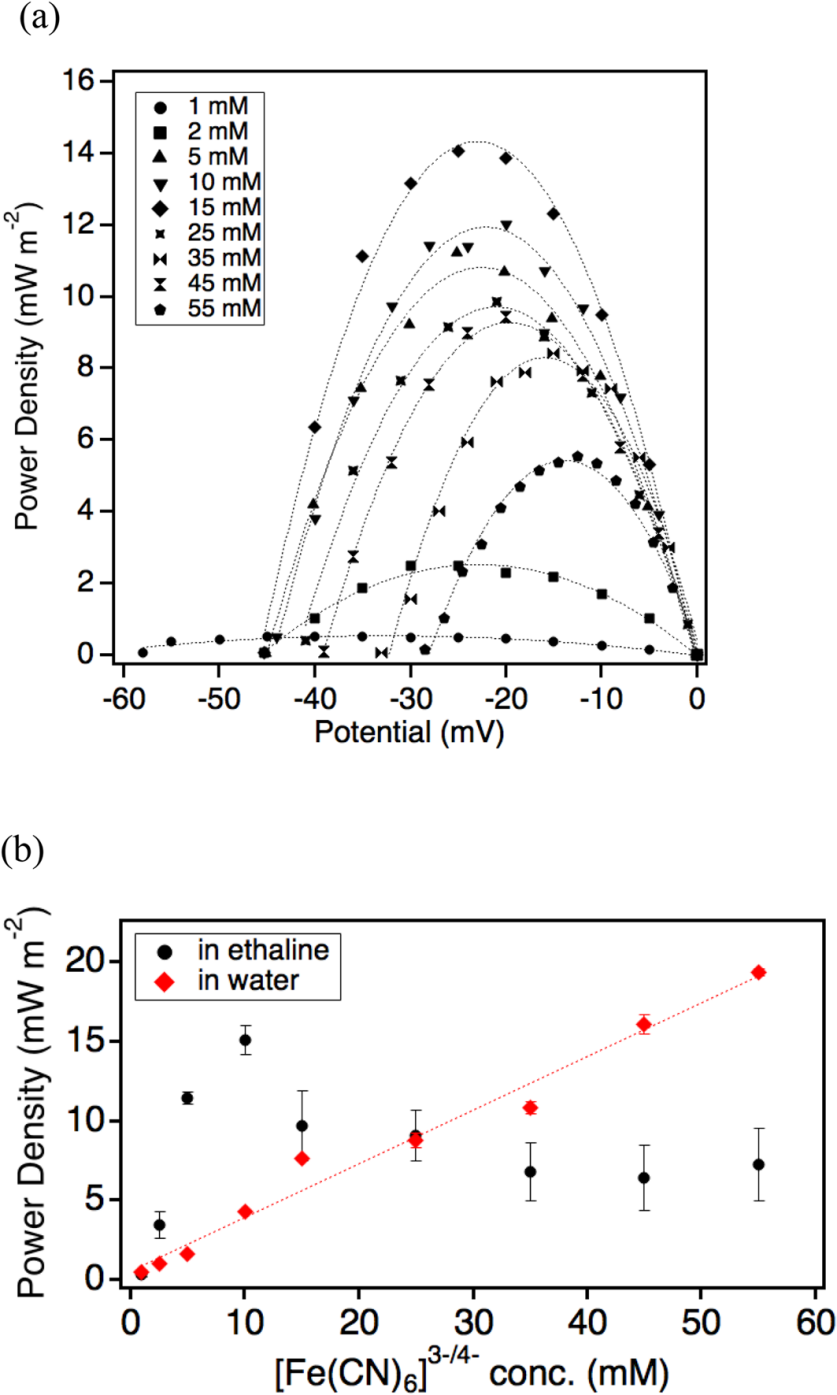
(Top) Power output of the ethaline thermocell (*T*_*hot*_ = ca. 165 °C, *T*_*cold*_ = 135.3 °C) and (bottom) change of maximum power output of the thermocell with increasing concentration of [Fe(CN)_6_]^3−/4−^ in ethaline (green) and water (orange) electrolytes.

The current–voltage relation of the ethaline thermocell was investigated to evaluate the power performance of the thermocell (Fig. 4). The *ΔT* across the electrolyte was maintained at 29.7 K, with the *T*_*ho*t_ = ca. 165 °C. The ethaline thermocell delivers a power density that is relatively higher than that of its aqueous counterpart, reaching 14 mW m^−2^ at its maximum, as shown in Fig. 4. This high power performance arises from the high ionic conductivity of the electrolyte due to the presence of charged components (Ch^+^ and Cl^−^) in the ethaline DES, as evaluated by the AC impedance method (Fig. S6). Furthermore, the high operating temperature also contributes to enhancing the ion transport across the cell^10–12^. The ethaline electrolyte displays low viscosity of less than 30 cP at *T* = 165 °C^19^ that allows for optimal ion transport and diffusion of the redox couple across the thermocell.

Interestingly, while the power output of ethaline thermocell increases with the increase of the concentration of redox couple, as is expected with the increase of the carriers, it displays a drop at the concentration of solutes above 25 mM. This drop might originate from the formation of ion pairs at high concentrations of charged species^26^ or the increased viscosity of electrolytes^12^. Such circumstances may impede mass transport which, in turn, decreases the power output of the thermocell. Nonetheless, the high *S*_*e*_ of the ethaline thermocell, which is higher than previously reported for non-aqueous thermocells^6,11^, demonstrates a potential for efficient thermoelectric conversion at middle-temperature ranges.

## Conclusion

In conclusion, we developed a novel thermocell system, which consists of [Fe(CN)_6_]^3−/4−^ redox couple and utilizes ethaline DES as an electrolyte. The thermocell displays a high Seebeck coefficient of − 1.67 mV K^−1^ and this value was obtained at 15 mM concentration of the respective solutes. This high S_e_ value is attributed to the large molar entropy difference between the redox species, which was investigated through FT-IR spectroscopic studies. Furthermore, the ethaline thermocell displays a high power output, which alludes to the overall high-performance efficiency of this novel thermocell system. This high Seebeck coefficient and operating-temperature flexibility demonstrate the prospect of utilizing DES-based thermocells for the recovery of middle-temperature waste heat.

## Methods

### Thermocell measurement

Various concentrations of K_3_[Fe(CN)_6_] and K_4_[Fe(CN)_6_] (1:1 in mol/mol) were dissolved into ethaline, and the electrolytes were mixed using a sonicator for ca. 3 h before use and heated above 150 °C before every measurement to ensure the removal of trace water. The solution was introduced into an H-shaped glass cell (diameter = 1.5 cm; electrode-electrode distance = 9 cm). The S_e_ measurement was conducted above 100 °C, and so aluminum heating-blocks were used to control the temperatures of the cold and hot sides. Platinum electrodes (∅ = 1 mm, h = 16.5 mm, working area = 52.62 mm^2^) were immersed into both sides of the thermocell and were connected to a source meter (Keithley 2401) to measure the open-circuit voltage (*V*_*oc*_) of the cell. The temperature of the thermocell was monitored by a probe thermocouple sensor (AS ONE TM201). During the measurement, the hot side temperature was kept at approximately 165 °C. The temperature of the cold side was increased gradually, and the *V*_*oc*_ was plotted against the temperature difference at which it was observed. The S_e_ of the ethaline thermocell was estimated from the slope of the plot.

The *S*_*e*_ measurements of the aqueous thermocell followed the same setup and method as those for the ethaline thermocell. Here, the cold side temperature is fixed at 10 °C (by using an ice water bath), and the hot side temperature is increased gradually to plot the *V*_*oc*_ at different temperature differences. Similarly, the S_e_ of the aqueous thermocell was estimated from the slope of the plot.

### Power and current performance measurement

The power and current performance of the ethaline thermocell were evaluated using the same setup and instruments as that of the *S*_*e*_ measurement, as shown above. The temperature difference between the two electrodes was kept at 29.7 °C, with the hot temperature set at approximately 165 °C and the cold temperature at 135.3 °C. The measurement was conducted by recording the current generated by various potential differences, namely *V*_*oc*_ to zero. The current density was determined by dividing the recorded current values by the working area of the platinum electrode (52.62 mm^2^). Subsequently, the power density generated by the thermocell was calculated by multiplying the current density by the recorded voltage. This data was then plotted against voltage and the maximum power output could be determined.

The power and current performance of the aqueous thermocell utilized the same setup as described for the ethaline thermocell. To maintain analogous experimental conditions to that of the ethaline thermocell, here the temperature difference was also kept at 29.7 °C (T_hot_ = 39.7 °C, T_cold_ = 10 °C). Other conditions (electrode are, measurement methods, etc.) were identical to those described above.

## Supplementary Information

Supplementary Information.
