# Supplementary material for: High seebeck coefficient in middle-temperature thermocell with deep eutectic solvent
Source: Sci Rep. 2021 Jun 7;11:11929. doi: 10.1038/s41598-021-91419-5 (PMC8184835; doi:10.1038/s41598-021-91419-5)
Supplement: Supplementary file 1 — Supplementary Information. [file 41598_2021_91419_MOESM1_ESM.docx]

**Supplementary Information**

**High Seebeck Coefficient in Middle-Temperature Thermocell with Deep Eutectic Solvent**

**Naura Fakhira Antariksa,^1^ Teppei Yamada,*^123^, Nobuo Kimizuka^12^**

^1^ Division of Chemistry and Biochemistry, Faculty of Engineering, Kyushu University, Motooka 744, Nishi-ku, Fukuoka 819-0395, Japan.

^2^ Center for Molecular Systems, Kyushu University.

^3^ Department of Chemistry, Graduate School of Science, The University of Tokyo, 7-3-1 Hongo, Bunkyo-ku, Tokyo 113-0033, Japan.

**Reagents information.** Choline chloride (ChCl) and ethylene glycol (EG) were purchased from Tokyo Chemical Industry (Japan). Potassium ferrocyanide trihydrate (K_4_Fe(CN)_6_.3H_2_O) and potassium ferricyanide (K_3_Fe(CN)_6_) (99 %) were purchased from Wako and Kishida (Japan), respectively. All reagents were used without further purification.

**Preparation of ethaline.** Ethaline DES was synthesized according to a previously reported method.^1–3^ Choline chloride (0.085 mol, 12.48 g) was mixed with ethylene glycol (0.17 mol, 10 mL) in a round-bottom flask. The mixture was stirred at 80 °C until a colorless liquid was formed. The product is kept in a tightly sealed bottle. ^1^H NMR in D_2_O:  δ 3.92 (m, 2H), 3.52 (s, 8H), 3.95 (m, 2H), 3.38 (t, 2H), 3.06 (s, 9H).

**Viscosity measurement.** The viscosity of ethaline DES was measured at room temperature. Before every measurement, the viscosity of methanol was taken as reference material to confirm the validity of the measurement. The sample was introduced to a glass bottle and the rotating probe was immersed in the sample. The viscosity of the sample was automatically determined by the instrument. The viscosity of ethaline DES was measured to be 51.1 cP at room temperature.

**DSC measurement.** The DSC curve of ethaline was measured using the DSC1 STARe system (METTLER TOLEDO) under N_2_ atmosphere. The sample was mounted on a 40 μL aluminum pan, and the measurement was conducted in one heating and cooling cycle (the heating rate applied was 0.2 K/min) from −80 to −40 °C. The low temperature was maintained using liquid nitrogen. The DSC traces of ethaline displays an exothermic peak at -68.9 °C, signifying released heat; in other words, the freezing point of the solvent. This result agrees with those reported in previous studies.^4,5^


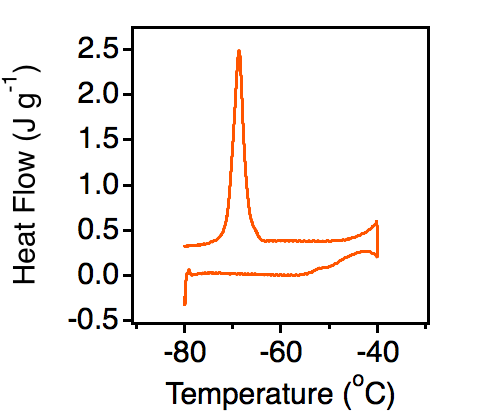


**Figure S1.** A DSC curve of ethaline.

**TG measurement.** TG analysis of DES ethaline was performed using the Thermo Plus EVO2 TG-DTA system under N_2_ gas flow. The sample was mounted on a 40 μL aluminum pan, and the measurement was conducted from 25 to 500 °C (the heating rate applied was 5 K/min). The TG curve has a couple of weight-loss regions, the first one attributed to the loss of water (around 70 to 100 °C), whereas the second weight loss corresponds to the decomposition of DES ethaline at around 210 °C.

**
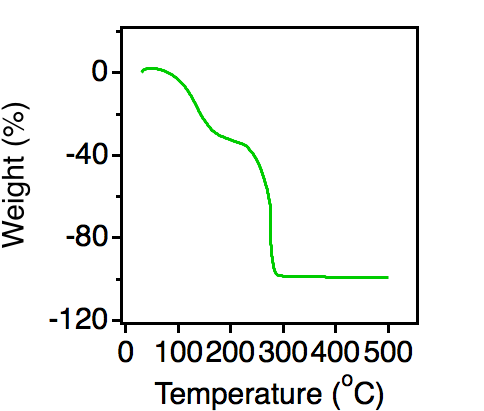
**

**Figure S2.** The thermogravimetric curve of ethaline.

**FT-IR measurement**. FT-IR measurements of the thermocell electrolyte was performed using IR Tracer-100 (Shimadzu). All the measurements were carried out using a demountable liquid cell (PIKE) composed of CaF_2_ windows and a 25 µm spacer. Each measurement was conducted from 1900 to 2500 cm^-1^ with 1 cm^-1^ resolution, mirror speed = 2.8, and at room temperature. All of the samples were dried on a heating block and sealed tightly before each measurement to minimize water exposure. The line shape analyses of the spectral data were conducted using IgorPro 7.

**Power and current performance measurement.** The power and current performance of the ethaline thermocell were evaluated using the same setup and instruments as that of the *S_e_* measurement, as shown above. The temperature difference between two electrodes was kept at 29.7 °C, with the hot temperature set at approximately 165 °C and the cold temperature at 135.3 °C. The measurement was conducted by recording the current generated by various potential differences, namely *V_oc_* to zero. The current density was determined by dividing the recorded current values by the working area of the platinum electrode (52.62 mm^2^). Subsequently, the power density generated by the thermocell was calculated by multiplying the current density to the recorded voltage. This data was then plotted against voltage and the maximum power output could be determined.

The power and current performance of the aqueous thermocell utilized the same setup as described for the ethaline thermocell. To maintain analogous experimental conditions to that of the ethaline thermocell, here the temperature difference was also kept at 29.7 °C (T_hot_ = 39.7 °C, T_cold_ = 10 °C). Other conditions (electrode are, measurement methods, etc.) were identical to that described above.

**Ionic conductivity of the ethaline electrolyte.** The ionic conductivity of the electrolyte was evaluated through electrochemical impedance spectroscopy. Approximately 5 mL of the standard solution was introduced to a glass bottle and two platinum wires (*φ* = 1 mm) were immersed into the solution. Thermocell electrolytes using ethaline were prepared by dissolving various concentrations of K_3_[Fe(CN)_6_] and K_4_[Fe(CN)_6_] (1:1 in mol/mol) into the solvent. The ethaline electrolytes were mixed using a sonicator for ca. 3 h before use and heated above 150 °C before every measurement to ensure the removal of trace water. The impedance measurements were carried out at the temperature range between 110 and 165 °C using a hot plate.

The two wires were kept apart at strictly 2 cm to maintain the cell constant (**Fig. 1.3**). The wires were then connected to an impedance instrument (Solartron 1296/1260). The impedance of the standard solution was measured at 1.000 kHz and the cell parameter was estimated according to the following formula:

$$\sigma=\frac{1}{R}\cdot C$$

where *σ* is the ionic conductivity, *1/R* is the inverse of impedance, and *C* is the cell parameter. 0.1 M KCl was used as a standard solution with a known conductivity (12.88 mS/cm), and the cell parameter C was estimated to be 907.2, which was used for evaluating the *σ*.


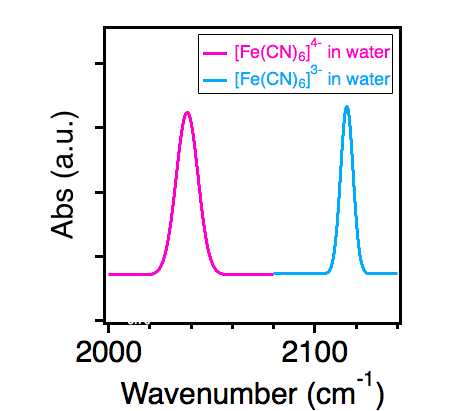

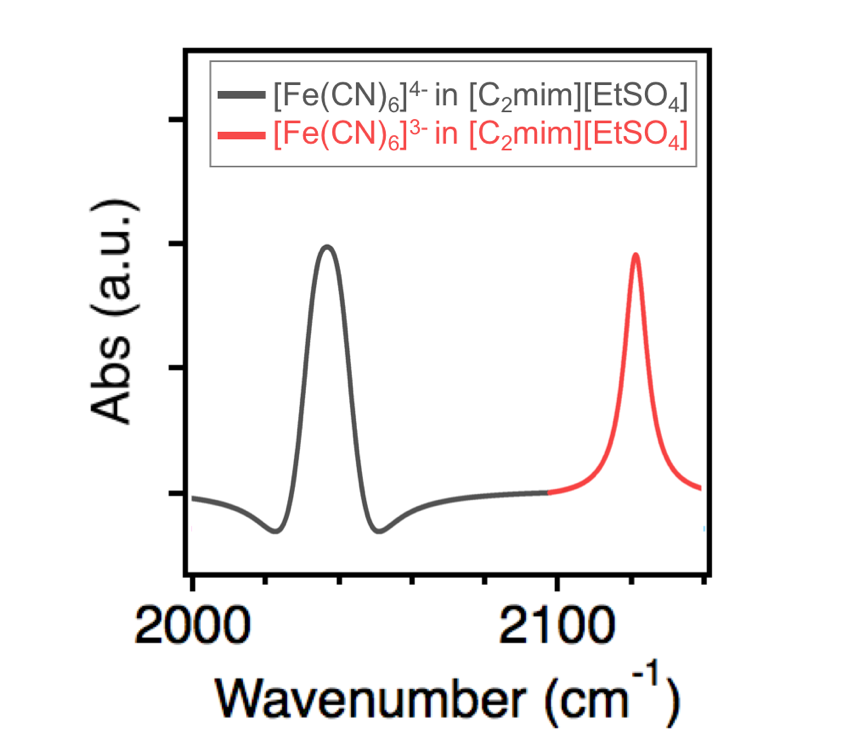

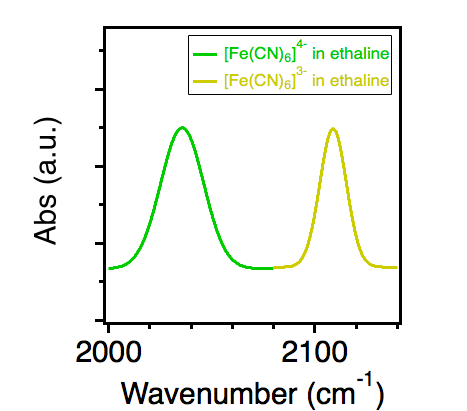


(c)

(b)

(a)

**Figure S3.** The FT-IR spectra of [Fe(CN)_6_]^4−^ and [Fe(CN)_6_]^3−^ in various solvents, displaying the C≡N stretching modes of the redox couple in ethaline (a), water (b), and [C_2_mim][EtSO_4_] (c). All redox couple concentrations were 15 mM. The spectral data were fitted using the pseudo-Voight function.^6^

**
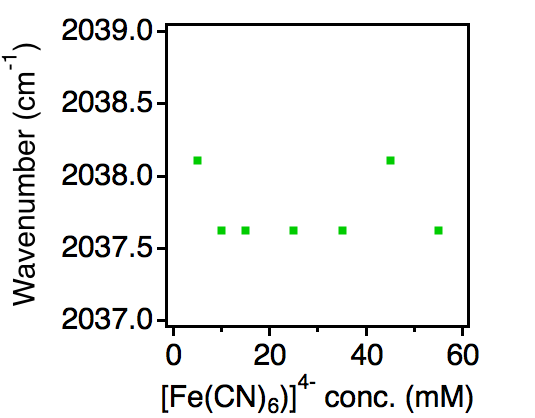
**

**
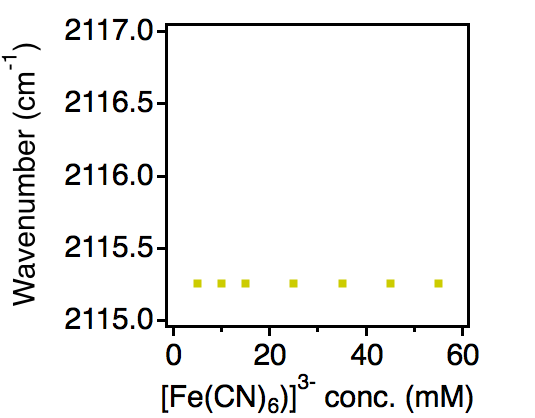
**

**Figure S4.** The peak wavenumbers of the C≡N stretching modes in [Fe(CN)_6_]^4−^ (top) and [Fe(CN)_6_]^3−^ (bottom) in water.


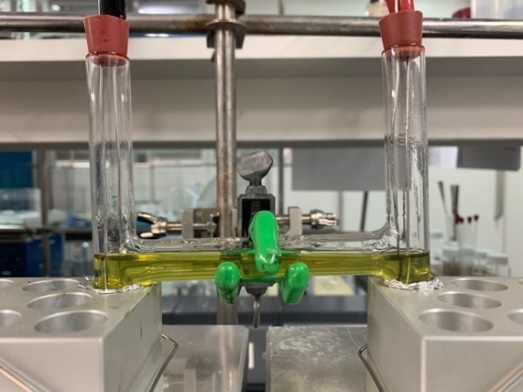

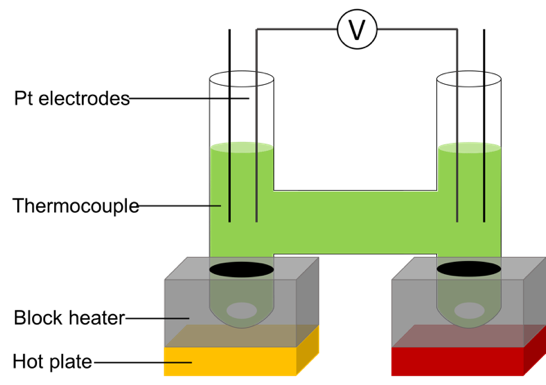


**Figure S5.** (Left) Schematic illustration of the H-shaped cell. (Right) Photo of the experimental setup of the *S_e_* measurement.

**
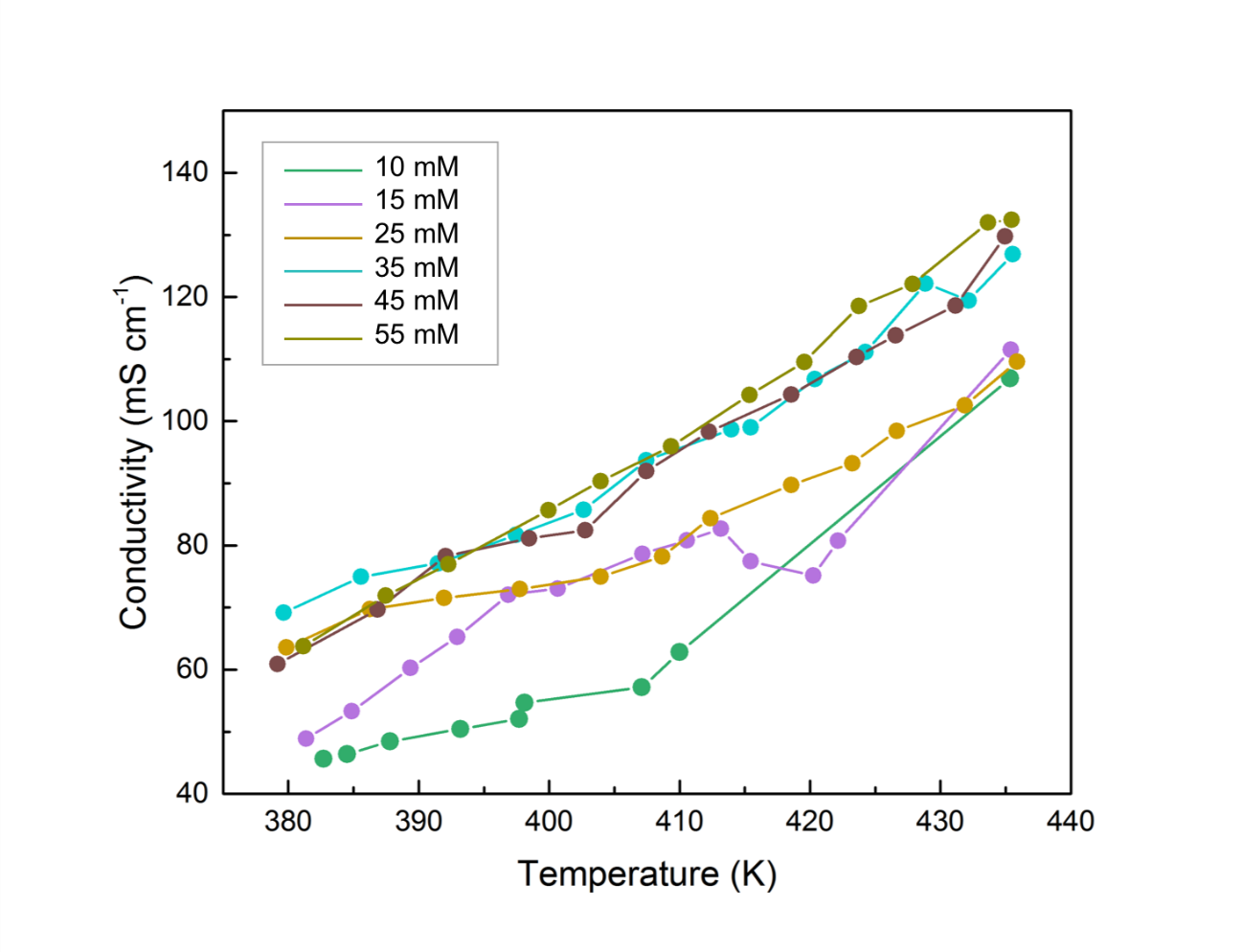
**

**Figure S6.** Electrical conductivity of ethaline electrolyte at different concentrations of redox couple at varied temperatures.

**Table S1.** Fitted parameters of C≡N absorption band of [Fe(CN)_6_]^3−/4−^ at 15 mM in various solvents

| redox species | solvent | FWHM / cm^−1^ | central frequency/cm^−1^ | *m* _lorentz_ |
| --- | --- | --- | --- | --- |
| [Fe(CN)_6_]^3−^ | ethaline | 22.5 | 2035.8 | −0.6 |
|  | water | 12.6 | 2038.7 | −0.1 |
|  | [C_2_mim][EtSO_4_] | 13.1 | 2043.2 | −7.4 |
| [Fe(CN)_6_]^4−^ | ethaline | 9.8 | 2108.8 | −0.1 |
|  | water | 7.4 | 2115.7 | 0.8 |
|  | [C_2_mim][EtSO_4_] | 8.3 | 2136.4 | 1.0 |

**Table S2.** The *S_e_* value of aqueous thermocells compared to its ionic liquid or molecular solvent counterparts

| Redox couple | Solven | \|*S_e_*\| (mV/K) |
| --- | --- | --- |
| [Fe(CN)_6_]^4-/3-^ | aqueous | 1.4 |
|  | BMPTFSA | 1.49 ± 0.05^8^ |
| [I_3_^−^]/[I^−^] | aqueous | 0.53 ± 0.05^7^ |
|  | methylpropionitrile | 0.26 ± 0.01^7^ |
|  | [C_2_mim][BF_4_] | 0.26 ± 0.01^7^ |
|  | [C_2_mim][NTf_2_] | 0.154 ± 0.005^7^ |
|  | ﻿[P_2,2,2,(101)_][NTf_2_] | 0.03 ± 0.01^7^ |

**Table S3.** The *S_e_* value obtained in this work (concentration of [Fe(CN)_6_]^3−/4−^ = 15 mM) compared to other studies on high-temperature thermocells.

| Electrolyte | *T*_hot_ (°C) | \|*S_e_*\| (mV/K) |
| --- | --- | --- |
| This work | ca. 160 | 1.67 |
| [C_2_mim][BF_4_] + [I_3_^−^]/[I^−^]^7^ | ca. 70 | 0.26 |
| [C_2_mim][NTf_2_] + [I_3_^−^]/[I^−^]^7^ | ca. 70 | 0.154 |
| [C_4_dmim][NTf_2_] + Co^II/III^(bpy)_3_(NTf_2_)]_2/3_^9^ | N/A | 1.32 |

**Table S4.** The list of *S_e_* values at various concentrations of ferrocyanide and ferricyanide and their measurement conditions.

| Ferrocyanide and ferricyanide | *T*_hot_ | Maximum *ΔT* | Minimum *ΔT* | *T*_cold_ range |
| --- | --- | --- | --- | --- |
| 1 mM | 165 | 29.3 | 5.3 | 135.7 – 159.7 |
| 2.5 mM | 165 | 40.6 | 0 | 124.4 – 165 |
| 5 mM | 165 | 38.2 | 10.8 | 126.8 – 154.2 |
| 10 mM | 165 | 41.3 | 8.2 | 123.7 – 156.8 |
| 15 mM | 165 | 32 | 3.1 | 133 – 161.9 |
| 25 mM | 165 | 47.3 | 5.7 | 117.7 – 159.3 |
| 35 mM | 165 | 29.9 | 2.3 | 135.1 – 162.7 |
| 45 mM | 165 | 43.7 | 7.7 | 121.3 – 157.3 |
| 55 mM | 165 | 46.4 | 14.4 | 118.6 – 150.6 |

**References**

1 A. Renjith and V. Lakshminarayanan, *J. Phys. Chem. C*, 2018, **122**, 25411–25421.

2 N. Frenzel, J. Hartley and G. Frisch, *Phys. Chem. Chem. Phys.*, 2017, **19**, 28841–28852.

3 A. Li, W. Duan, J. Liu, K. Zhuo, Y. Chen and J. Wang, *Sci. Rep.*, 2018, **8**, 13141.

4 G. García, S. Aparicio, R. Ullah and M. Atilhan, *Energy and Fuels*, , DOI:10.1021/ef5028873.

5 D. Reuter, C. Binder, P. Lunkenheimer and A. Loidl, *Phys. Chem. Chem. Phys.*, , DOI:10.1039/c9cp00742c.

6 M. G. Maienschein-Cline and C. H. Londergan, *J. Phys. Chem. A*, 2007, **111**, 10020–10025.

7 T. J. Abraham, D. R. MacFarlane and J. M. Pringle, *Chem. Commun.*, 2011, **47**, 6260–6262.

8 T. Migita, N. Tachikawa, Y. Katayama and T. Miura, *Electrochemistry*, 2009, **77**, 639–641.

9 J. He, D. Al-Masri, D. R. MacFarlane and J. M. Pringle, *Faraday Discuss.*, 2016, **190**, 205–218.
